# Cost-effectiveness analysis of voice rehabilitation for patients with laryngeal cancer: a randomized controlled study

**DOI:** 10.1007/s00520-020-05362-8

**Published:** 2020-02-20

**Authors:** Mia Johansson, Caterina Finizia, Josefine Persson, Lisa Tuomi

**Affiliations:** 1 Department of Oncology, Institute of Clinical Sciences, Sahlgrenska Academy at the Gothenburg University, Gothenburg, Sweden; 2 Department of Oncology, Sahlgrenska University Hospital, Region Västra Götaland, Gothenburg, Sweden; 3 Department of Otorhinolaryngology, Head and Neck surgery, Institute of Clinical Sciences, Sahlgrenska Academy, Gothenburg University, Gothenburg, Sweden; 4 Department of Otorhinolaryngology, Sahlgrenska University Hospital, Region Västra Götaland, Gothenburg, Sweden; 5 Health Economics and Policy, School of Public Health and Community Medicine, Institute of Medicine, University of Gothenburg, Gothenburg, Sweden

**Keywords:** Laryngeal neoplasms, Voice therapy, Costs, Cost-effectiveness, Radiotherapy, Quality of life

## Abstract

**Introduction:**

Voice problems are common following radiotherapy for laryngeal cancer. Few studies exist covering the effect of voice rehabilitation, and no previous studies exist regarding the cost of said rehabilitation. This randomized controlled study aimed to analyze the cost-effectiveness of voice rehabilitation after radiotherapy for patients with laryngeal cancer.

**Material and methods:**

A total of 66 patients with laryngeal cancer with follow-up data 12 months post-radiotherapy were included. Patients were randomized into receiving either voice rehabilitation (*n* = 32) or no voice rehabilitation (*n* = 34). The patient outcome was measured as quality-adjusted life years (QALYs). The index range between 0 and 1, where 0 equals death and 1 represents perfect health. The QALYs were assessed with the European Organization for Research and Treatment of Cancer questionnaire QLQ-C30 mapped to EuroQoL 5 Dimension values. The cost of rehabilitation and other healthcare visits was derived from hospital systems. The patients reported the total amount of sick leave days during the first 12 months following radiotherapy. The cost-effectiveness of the voice rehabilitation was compared with no rehabilitation intervention based on the incremental cost-effectiveness ratio.

**Results:**

The cost per gained QALY with voice rehabilitation compared to no rehabilitation from a societal perspective was − 27,594 € (SEK − 250,852) which indicates that the voice rehabilitation is a cost-saving alternative compared to no rehabilitation due to lower costs and a slightly better health outcome. From a healthcare perspective, the voice rehabilitation indicates a cost 60,800 € (SEK 552,725) per gained QALY.

**Conclusion:**

From a societal perspective, i.e., including the costs of production loss, voice rehabilitation compared to no voice rehabilitation following radiotherapy for laryngeal cancer seems to be cost-saving. When analyzing only the healthcare costs in relation to health outcomes, voice rehabilitation indicates an incremental cost of 60,800 € per gained QALY, which is just above the threshold of the maximum willingness to pay level.

## Introduction

Vocal dysfunction is a common side effect following radiotherapy for laryngeal cancer [1–3]. Studies show that voice problems occur in a majority of patients (40–100%) and may persist up to 10 years following completion of radiotherapy [4–7]. The voice problems are often explained by reductions of the mucosal wave, scarring in the vocal fold tissue, inelasticity, and sometimes glottic inadequacy, which might result in compensatory behaviors in voice production [1, 3]. The changes can affect the audible sound or lead to a feeling of troublesome voice production, both which may lead to a disrupted social life [8]. Therefore, voice rehabilitation has been suggested and assumed helpful for this patient group [9–11]. Few studies exist where the efficacy of voice rehabilitation for laryngeal cancer patients has been evaluated, but the studies that exist demonstrate a positive effect on voice quality, voice function, and health-related quality of life (HRQL) [5, 12–14]. Recently, our research group has reported the effects of voice rehabilitation in a randomized controlled study with follow-up up to 12 months following radiotherapy [15–20]. Voice rehabilitation improved health related quality of life, communicative function, and prevented the deterioration of voice quality over time, effects that remain 12 months post-radiotherapy. Even though positive effects have been demonstrated, no account has been made regarding the costs of said treatment. In order to implement the research results in clinical praxis, the cost of voice rehabilitation in relation to the patient’s health effect need to be addressed. To the authors’ knowledge, no studies assessing the cost-effectiveness of voice therapy exist. The objective of this study was therefore to analyze the cost-effectiveness of voice rehabilitation after radiotherapy for patients with laryngeal cancer.

## Material and methods

### Participants

All patients diagnosed with laryngeal cancer in the Region Västra Götaland are discussed on a weekly multidisciplinary tumor board, where treatment options are discussed and decided for each patient. Between the years 2000 and 2011, with an interruption of 2 years, the patients who were to receive curatively intended radiotherapy ± chemotherapy were asked to participate in the study. Inclusion criteria were sufficient cognitive ability and sufficient knowledge of the Swedish language in order to fill out study questionnaires. Comorbidity was measured with the Adult Comorbidity Evaluation (ACE-27) [21, 22].

### Study design

The computerized randomization followed Pocock’s sequential randomization method [23] for optimal allocation regarding age, gender, smoking habits, tumor site, tumor size, and patients’ self-evaluation of communication [24]. Sample size was determined by an 80% power calculation, with dysphonia as the main variable. The groups were set to be of equal size. The power calculation prescribed a total of 80 patients, including an expected drop-out of ten patients. The voice rehabilitation group received voice rehabilitation after the completion of radiotherapy (between the 1-month and 6-month follow-up), while the control group received general vocal hygiene advice according to clinical practice. Both groups were followed up with audio recordings and questionnaires at similar time-points. The follow-ups were made at 1, 6, and 12 months post-radiotherapy.

Voice rehabilitation was given between 1 and 6 months post-radiotherapy, by speech-language pathologists. The rehabilitation protocol consisted of 10 occasions over the course of 10 weeks, approximately 30 min per rehabilitation session. The sessions included a specified set of exercises of breathing, relaxation, and phonation exercises, both indirect and direct therapy techniques. The patients were encouraged to exercise at home between sessions. The protocol has been described in detail elsewhere [18].

### Oncological treatment

Radiotherapy was given in accordance with regional treatment guidelines, either as conventionally fractioned (*n* = 45) or hyperfractioned (*n* = 21). Conventionally, fractionated therapy was given once daily in 2–2.4 Gy fractions to a total dose of 64.6–68 Gy. Hyperfractioned therapy entailed 1.7 Gy doses that were given twice daily to a total dose of 64.6 Gy. Most patients with T2-T4 tumors also received irradiation to regional lymph nodes to a total dose of 40.8–46 Gy. Three patients received chemotherapy in addition to radiotherapy.

### Quality-adjusted life years

The patient outcome was measured as quality-adjusted life years (QALYs). QALY is a measurement that combines health-related quality of life (HRQL) and life expectancy in one index. The index range between 0 and 1, where 0 equals death and 1 represents perfect health. Thus, 1 QALY corresponds to 1 year of perfect health [25]. Life expectancy is not affected by the voice rehabilitation, why any difference in the gained QALYs is due to an increase in HRQL, often called QALY-weights. The QALY-weights weres assessed with the European Organization for Research and Treatment of Cancer (EORTC) questionnaire QLQ-C30 mapped to EuroQoL 5 Dimensions (EQ-5D) values by Proskorovsky et al. [26]. The EORTC QLQ-30 is a validated 30-item questionnaire [27]. The questionnaire includes five functional scales (Physical, Role, Cognitive, Emotional, and Social Functioning), three symptom scales (Fatigue, Pain, and Nausea/Vomiting), a Global Health/HRQL scale, and six single items (Constipation, Diarrhea, Insomnia, Dyspnea, Appetite Loss, and Financial Difficulties). EQ-5D is a self-administered questionnaire consisting of five dimensions (Mobility, Self-Care, Pain, Usual Activities, and Anxiety/Depression) with three levels in each dimension (none, moderate and severe problems). In the mapping algorithm by Proskorovsky et al. [26], the EQ-5D health states were converted into a single health index using the UK value sets [28]. In the base case analysis, the full algorithm model by Proskorovsky et al. [26] was used, i.e., all the items in the EORTC QLQ-C30 questionnaire were used to map the EQ-5D QALY-weights. The EORTC QLQ-C30 was assessed 1, 6, and 12 months after radiotherapy. QALYs were estimated individually for each patient as the area under the curve by using time-weighted QALY-weights for the time spent in each health state [25]. Thus, the QALYs were estimated reflecting the change during the period between 1, 6, and 12 months post-radiotherapy. The total QALYs for each alternative were estimated as the means of the time-weighted individual QALYs.

### Costs

The costs were categorized as either direct healthcare costs or loss of production. The cost of cancer treatment is not included in the analysis. All costs regarding hospital admission days and hospital visits over the 12 months following radiotherapy were derived from the hospital charts and hospital administration and were calculated for each patient. The loss of production was based on the number of sick leave days after radiotherapy treatment and during 1-year follow-up. The loss of production was valued by the human capital approach assuming that production loss is valued at market price [29], i.e., gross salaries and payroll taxes. A daily estimation including payroll taxes of 215 € (SEK 1950) [30] was used. The cost of sick leave was calculated for the able-bodied patients, i.e., the patients that were working at the time of the study (*n* = 26), where the patients were asked about the amount of sick leave during the 12 months following radiotherapy.

Costs are presented in euros (€/EUR) and in Swedish kronor (SEK) using 2013 exchange rates for conversion to Swedish kronor (SEK 8.98 = 1 EUR).

### Cost-effectiveness analysis

The cost-effectiveness of the voice rehabilitation was compared with no rehabilitation intervention based on the incremental cost-effectiveness ratio (ICER). The ICER is defined by the difference in cost between the two alternatives, divided by the difference in their effects. Thus, the ICER could be interpreted as the incremental cost associated with 1 additional QALY. The ICER is calculated as follows:

$$ \mathrm{ICER}=\frac{{\mathrm{Cost}}_{\mathrm{voice}\ \mathrm{rehabilitation}}-{\mathrm{Cost}}_{\mathrm{no}\ \mathrm{rehabilitation}}}{{\mathrm{QALYs}}_{\mathrm{voice}\ \mathrm{rehabilitation}}-{\mathrm{QALYs}}_{\mathrm{no}\ \mathrm{rehabilitation}}} $$

The cost-effectiveness analyses are presented from two perspectives, the societal perspective (including both direct healthcare costs and loss of production), and the healthcare perspective (including direct healthcare costs). The costs and health outcomes were not discounted due to a 1-year time horizon.

### Assessing uncertainties

**Fig. 1:**
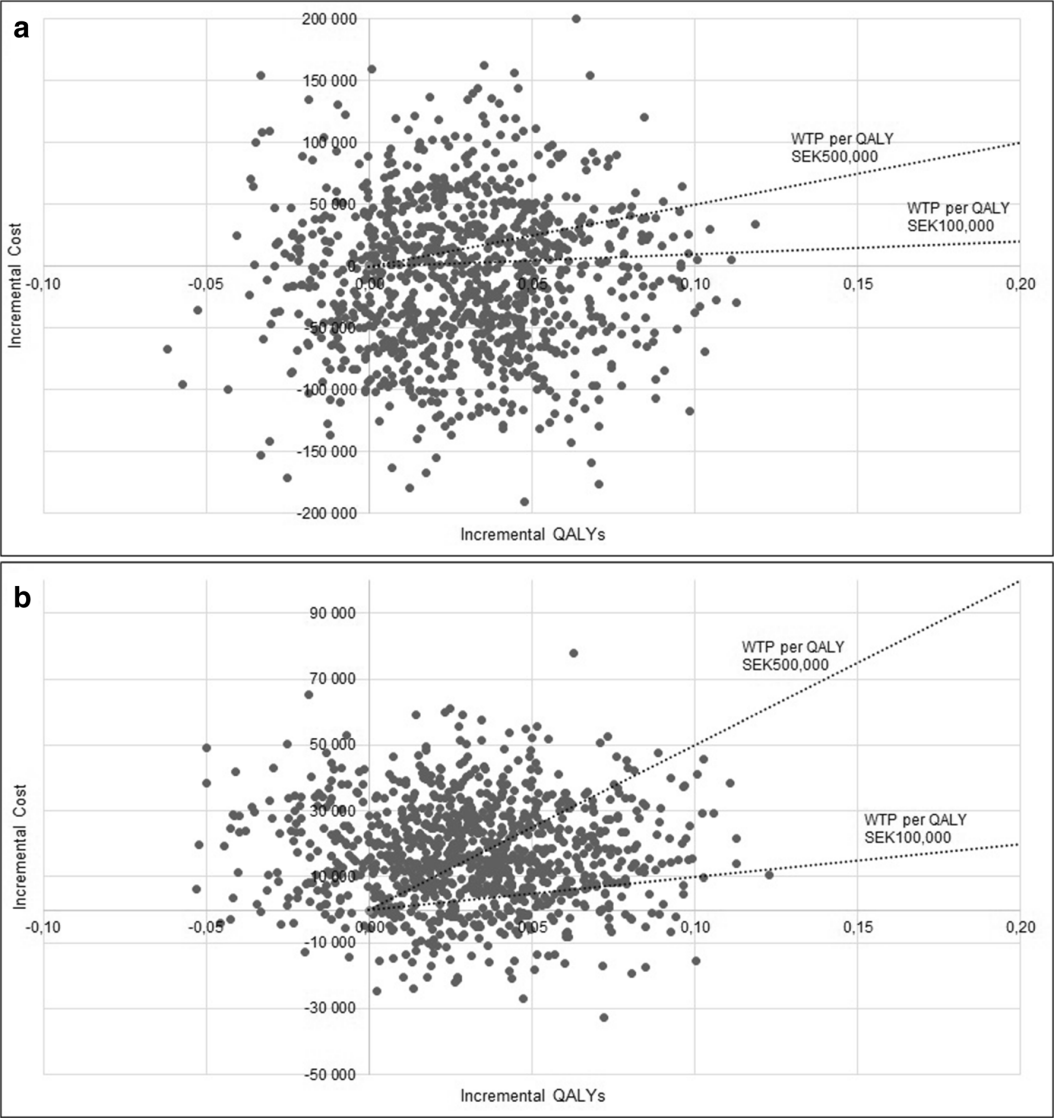
Cost-effectiveness plane **a** in a societal perspective and **b** in a healthcare perspective

A non-parametric bootstrapping was conducted to demonstrate the uncertainties surrounding the ICER. The bootstrapping was conducted using 1000 bootstrap replicates. The results of the bootstrapping are shown using a cost-effectiveness plane in Fig. 1. The cost-effectiveness plane graphically plots the differences in costs and QALYs between voice rehabilitation compared to no rehabilitation. No rehabilitation is plotted at the origin of the graph, and the incremental costs and QALYs with voice rehabilitation are represented at the *x*- and *y*-axes. The maximum willingness to pay was set to 55,000 € (SEK 500,000) according to the Swedish National Board of Health and Welfare [31].

There is no existing algorithm for mapping EORTC QLQ-C30 to EQ-5D values for patients with laryngeal cancer; thus, a deterministic sensitivity analysis was carried out with the existing algorithms for other types of cancer [32–35].

### Statistical analysis

The variable distribution was presented as mean and standard deviation (95% CI) for continuous variables and as number and percentage for categorical variables. All significance tests were two-sided and conducted at the 5% significance level. For comparison between groups, the non-parametric Mann-Whitney *U* test was used due to skewed data. All the analyses were carried out in SPSS software (version 25, SPSS, Inc., Chicago, IL, USA).

## Results

A total of 194 patients were assessed for eligibility in the study, and 163 patients met the inclusion criteria. Of these, 89 patients (55%) chose to participate in the study and were allocated to voice rehabilitation group or control group. Twelve patients discontinued their participation before their first follow-up, leaving a total of 77 patients (87%). An additional 11 patients discontinued their participation before the 12-month follow-up, leaving 32 patients in the voice rehabilitation group and 34 in the control group. Reasons for discontinuation were missed appointment (*n* = 2), laryngectomy (*n* = 4), and patient choice (*n* = 5).

The patient characteristics of included patients are listed in Table 1. No statistically significant changes were found between the groups regarding age, sex, tumor location, tumor stage, comorbidity, or type of treatment. A total of 26 patients (39%) were considered able-bodied, i.e., were working at the time of the study, and included in the analysis of the societal perspective.

**Table 1:** Patient characteristics

	Voice rehabilitation *n* = 32	No voice rehabilitation *n* = 34	*p* value
Mean age (SD)	64.8 (13.0)	62.5 (10.1)	ns
	**n (%)**	**n (%)**	
Sex			ns
Male	28 (87.5)	30 (88)	
Female	4 (12.5)	4 (12)	
Smoking habits 1 year post-radiotherapy			ns
Smoker	6 (19)	2 (6)	
Non-smoker	7 (22)	9 (26)	
Quit smoking > 12 months ago	19 (59)	23(68)	
Tumor location			ns
Glottis	26 (81)	25 (73.5)	
Supraglottis	6 (19)	8 (23.5)	
Subglottis	0	1 (3)	
Tumor stage			ns
0	0 (0)	1 (3)	
I	22 (69)	17 (50)	
II	8 (25)	11 (32)	
III	1 (3)	5 (15)	
IV	1 (3)	0 (0)	
Comorbidity 1 year post-radiotherapy (ACE-27)			ns
None	14 (44)	14 (41)	
Mild	9 (28)	15 (44)	
Moderate	9 (28)	4 (12)	
Severe	0 (0)	1 (3)	
Radiotherapy			ns
Conventional	23 (72)	22 (65)	
Hyperfractioned	9 (28)	12 (35)	
Chemotherapy	2 (6)	1 (3)	ns

*ACE-27* Adult Comorbidity Evaluation

Table 2 shows the mean number of visits to oncologists and otorhinolaryngologists, hospital admission days, and costs. The mean healthcare cost for the group with voice rehabilitation was 6775 € ranging from 2893 € to 41,802 € (SEK 61,594; range SEK 26,298 to SEK 380,017) and the mean healthcare cost for the group with no rehabilitation was 4945 € range from 453 € to 32,842 € (SEK 44,959; range SEK 4118 to SEK 298,868). There was a significant difference in the direct healthcare costs between the voice rehabilitation group and no rehabilitation group.

**Table 2:** Costs per patient during first year after radiotherapy presented in EUR (SD)

	Total (*n* = 66)	Voice rehabilitation (*n* = 32)	No rehabilitation (*n* = 34)	*p* value
Number of visits to oncologists	3.11 (0.98)	3.09 (0.76)	3.12 (1.15)	n.s.
Costs of visits to oncologists	712 (224)	709 (178)	715 (263)	n.s.
Number of hospital admission days to Oncology	0.64 (2.58)	0.59 (3.31)	0.68 (1.59)	n.s.
Costs of hospital admission to Oncology	509 (2066)	475 (2687)	541 (1272)	n.s.
Number of visits to otorhinolaryngologists	1.79 (1.93)	1.81 (2.40)	1.76 (1.37)	n.s.
Costs of visits to otorhinolaryngologists	493 (531)	500 (662)	486 (378)	n.s.
Number of hospital admission days to otorhinolaryngology	4.06 (9.64)	3.72 (9.72)	4.38 (9.70)	n.s.
Cost of hospital admission to otorhinolaryngology	2937 (6970)	2690 (7027)	3170 (7012)	n.s.
Number of other healthcare visits	0.23 (1.08)	0.06 (0.35)	0.38 (1.46)	n.s.
Costs of other healthcare visits	56 (266)	15 (87)	94 (360)	n.s.
Cost of voice rehabilitation	2470	2470	–	–
Total direct healthcare costs	5905 (7364)	6859 (7726)	5007 (7002)	< 0.001
Number of sick leave days	78.82 (111.36)	67.66 (107.44)	85.72 (116.01)	n.s.
Loss of production	16,079 (23,784)	14,692 (23,330)	17,385 (24,479)	n.s.
Total costs	21,984 (23,843)	21,551 (27,472)	22,391 (26,658)	n.s.

The mean cost due to loss of production for the group with voice rehabilitation was 14,512 €, with a range from 0 € to 72,072 € (SEK 131,930; range SEK 0 to SEK 655,200) and the mean healthcare cost for the group with no rehabilitation was 17,173 € with a range from 0 € to 65,422 € (SEK 156,115; range SEK 0 to SEK 594,750).

The QALYs were calculated as the area under the curve by using time-weighted QALY-weight taking account of the time spent in each health state (Table 3). The mean QALYs were 0.87 (range 0.53 to 1) and 0.84 (range 0.57 to 1) in the voice rehabilitation and no rehabilitation group, respectively, indicating a difference in QALYs of 0.03 points. The difference in health outcomes was not significant.

**Table 3:** Health outcomes based on the EORTC QLQ-C30 questionnaire mapped into EQ-5D QALY-weights (SD), comparisons between groups at each time-point, and mean time-weighted QALYs (95% CI)

	Total (*n* = 66)	Voice rehabilitation (*n* = 32)	No rehabilitation (*n* = 34)	*p* value
1 month after radiotherapy	0.80 (0.15)	0.81 (0.14)	0.79 (0.17)	n.s.
6 months after radiotherapy	0.88 (0.13)	0.89 (0.13)	0.86 (0.14)	n.s.
12 months after radiotherapy	0.88 (0.14)	0.90 (0.14)	0.86 (0.14)	n.s.
Mean time-weighted QALYs (95% CI)	0.86 (0.82–0.92)	0.87 (0.83–0.92)	0.84 (0.80–0.89)	n.s.

*QALY* quality-adjusted life year, *CI* confidence interval

The cost-effectiveness results are shown in Table 4. From a societal perspective, the cost per gained QALY with voice rehabilitation compared to no rehabilitation was − 27,594 € (SEK − 250,852). Thus, the voice rehabilitation is a cost-saving alternative compared to no rehabilitation due to lower costs and associated with a slightly better health outcome, and thus a dominant alternative. From a healthcare perspective, the voice rehabilitation indicates a cost of 60,800 € (SEK 552,725) per gained QALY, which is just above the threshold of the maximum willingness to pay level.

**Table 4:** Cost-effectiveness results

	Difference in QALYs (95% CI)	Difference in costs (95% CI)	ICER EUR/QALY	ICER SEK/QALY
Societal perspective				
Voice rehabilitation vs no rehabilitation	0.03 (− 0.03–0.09)	− 7550 (− 130,380–113,598)	− 27,594	− 250,852
Healthcare perspective				
Voice rehabilitation vs no rehabilitation	0.03 (− 0.03–0.09)	16,635 (− 15,402–50,772)	60,800	552,725

*QALY* quality-adjusted life year, *EUR* euro, *SEK* Swedish kronor, *ICER* incremental cost effectiveness ratio, *CI* confidence interval

### Sample uncertainty and sensitivity analysis

A non-parametric bootstrapping with 1000 bootstrap replicates was conducted to assess the uncertainty in the cost-effectiveness ratio. The results are presented in a cost-effectiveness plane (Fig. 1), which graphically plots the differences in costs and QALYs between voice rehabilitation compared to no rehabilitation.

From a societal perspective and at a threshold of 55,000 € (SEK 500,000), there is a 66% probability that voice rehabilitation is cost-effective compare to no rehabilitation. From a healthcare perspective and at a threshold of 55,000 € (SEK 500,000), there is a 62% probability that voice rehabilitation is cost-effective compared to no rehabilitation.

Table 5 shows the sample’s QALY weights using the different mapping algorithms indicating a difference in gained QALYs that ranges from 0.003 with the algorithm for breast and colorectal cancer to 0.030 with the algorithm for multiple myeloma. Thus, the ICER ranges from 61,500 € to 556,000 € (SEK 552,725 to SEK 5.1 million) when applying the different mapping algorithms.

**Table 5:** Health outcomes based on the EORTC QLQ-C30 questionnaire mapped into EQ-5D QALY-weights using different mapping algorithms

Type of cancer	Total (*n* = 66)	Voice rehabilitation (*n* = 32)	No rehabilitation (*n* = 34)	Difference in QALYs	*p* value	Source of algorithm
Multiple myeloma	0.86	0.87	0.84	0.03	n.s.	[21] Full model*
Multiple myeloma	0.84	0.86	0.83	0.03	n.s.	[21] Trimmed model
Inoperable esophageal	0.80	0.82	0.80	0.02	n.s.	[32] Full model
Gastric	0.60	0.63	0.60	0.03	n.s.	[26] EQ-5D
Gastric	0.69	0.70	0.69	0.01	n.s.	[26] SF-6D
Gastric	0.73	0.74	0.72	0.02	n.s.	[26] 15D
Non-small cell lung	0.82	0.82	0.81	0.01	n.s.	[33] Full model
Breast	0.95	0.82	0.81	0.01	n.s.	[24] Full model
Breast and colorectal	0.98	0.98	0.98	0.00	n.s.	[25] Full model

*Base case analysis

## Discussion

This study aimed to investigate the cost-effectiveness of a structured voice rehabilitation protocol following radiotherapy for laryngeal cancer. Previous studies have shown that the effects of voice rehabilitation include improved HRQL, which prevents a deterioration of perceived roughness and better self-perceived communicative function [17, 18, 20]. The present study showed that from a societal perspective, the voice rehabilitation is a cost-saving alternative compared to no rehabilitation due to lower costs and associated with a slightly better health outcome.

The gain from a societal perspective was the decreased number of sick leave days from an average of 85.72 with no rehabilitation to 67.66 with voice rehabilitation. The number of sick leave days in the group without voice rehabilitation is similar to a study by Cohen et al., where laryngeal cancer patients had a mean of 97.89 sick leave days over a 12-month period [36]. Although, due to the wide range in sick leave days in the study population, the difference in production loss between the two groups was not statistically significant. The wide range in the production loss is also the main factor for the sampling uncertainty, illustrated in Fig. 1a. The result from a healthcare perspective was 60,800 € with voice rehabilitation compared to no rehabilitation. This result was just above the threshold of 55,000 € set as a “rule-of-thumb” by the Swedish National Board of Health and Welfare, with 62% probability that the voice rehabilitation is cost-effective.

As previously stated, no mapping algorithm exists to convert EORTC QLQ-C30 data to utility scores for this patient group. The authors therefore looked at mapping algorithms for different patient groups, and the main algorithm chosen was the full model by Proskorovsky et al. [26]. This model was chosen since the age of the included patients as well as the EORTC QLQ-C30 values were similar to the present patient group. However, the prognosis for laryngeal cancer is generally better compared to the tumors stated in Table 5, which hinders comparison. Therefore, in order to perform further analyses, a mapping algorithm for the head and neck cancer population should be developed. Due to the lack of a mapping algorithm developed for this patient group, a sensitivity analysis performing the calculations with different algorithms was performed. This showed that the utility scores differed depending on which algorithm was used, subsequently affecting the ICER, which differed greatly (range from 61,500 € to 556,000 € in a healthcare perspective). Doble et al. 2016 [37] has assessed the external validity of the algorithms used in the deterministic sensitivity analysis resulted in a poor predictive accuracy. The result also indicated that the algorithms are insensitive to the grouping of tumor type and more sensitive to severity of the disease. Due to this, the authors recommend that an extensive scenario analysis should be conducted when the algorithms are used in cost-effectiveness analyses. The deterministic sensitivity analysis in this study confirms this statement resulting in a wide range of ICERs.

Additionally, the utility scores are mapped from the EORTC QLQ-C30, which might not be the most appropriate questionnaire to find the differences that are expected following voice rehabilitation. The EORTC QLQ-C30 is developed for measuring HRQL in cancer patients in general, and previously, the laryngeal cancer population has been found to demonstrate good HRQL at 1 year following treatment when using the EORTC QLQ-C30, with values comparable to the values of a normative population [38, 39]. However, when measured with tumor specific questionnaires, or questionnaires regarding communicative function, patients do report problems at 1 year following oncologic treatment [39, 40]. Therefore, the cost-effectiveness might be better calculated using another method, better corresponding to the changes that are expected to occur following voice rehabilitation, or should be completed with comparisons of effects in other domains such as communication and speech, for example, the speech domain of the EORTC QLQ head and neck module or the self-evaluation of communication experiences after laryngeal cancer [24, 40, 41].

The main strength with this cost-effectiveness analysis is that the data were collected prospective alongside a clinical trial and that the data are patient reported. A limitation to this study is the small patient sample with large variation of healthcare utilization and production loss, as well as that the power calculation for sample size was performed for voice outcomes only, and not costs and QALYs. Another limitation is that the sick leave days were derived mainly from the patients themselves, who were asked to state, in months, how much sick leave they had from work with increased risk of recall-bias. The number of sick leave day would probably be more accurate if this had been collected more precise, i.e., if the patient stated number of days or collected from registries. However, in this case, it was not possible, possibly leading to some bias regarding the total number of days of sick leave.

## Conclusion

Voice rehabilitation compared to no voice rehabilitation following radiotherapy for laryngeal cancer seems to be cost-saving in a societal perspective, i.e., when including both healthcare costs and the costs of production loss. When analyzing only the healthcare costs in relation to quality outcomes, voice rehabilitation indicates a cost per QALY which is just above the maximum willingness to pay level. Despite this, this study provides important information to decision makers regarding the benefits of voice rehabilitation in a societal perspective.
